# Supplementary material for: Neutralizing IFNγ improves safety without compromising efficacy of CAR-T cell therapy in B-cell malignancies
Source: Nat Commun. 2023 Jun 9;14:3423. doi: 10.1038/s41467-023-38723-y (PMC10256701; doi:10.1038/s41467-023-38723-y)
Supplement: Supplementary file 1 — Supplementary Information [file 41467_2023_38723_MOESM1_ESM.pdf]

# Neutralizing IFNγ improves safety without compromising efficacy of CAR-T cell therapy in B-cell malignancies

Simona Manni#[1], Francesca Del Bufalo#[1], Pietro Merli[1], Domenico Alessandro Silvestris[1], Marika Guercio[1], Simona Caruso[1], Sofia Reddel[1], Laura Iaffaldano[1], Michele Pezzella[1], Stefano Di Cecca[1], Matilde Sinibaldi[1], Alessio Ottaviani[1], Maria Cecilia Quadraccia[1], Mariasole Aurigemma[1], Andrea Sarcinelli[1], Roselia Ciccone[1], Zeinab Abbaszadeh[1], Manuela Ceccarelli[1], Rita De Vito[2], Maria Chiara Lodi[1], Maria Giuseppina Cefalo[1], Angela Mastronuzzi[1], Biagio De Angelis*[1], Franco Locatelli*§[1,3], Concetta Quintarelli§[1,4] .

1 Department of Haematology-Oncology and Cell and Gene Therapy, Bambino Gesù Children Hospital, IRCCS, Rome, Italy.
2 Department of Pathological Anatomy, Bambino Gesù Children Hospital, IRCCS, Rome, Italy.
3 Department of Pediatrics, Catholic University of the Sacred Heart, Rome, Italy.
4 Department of Clinical Medicine and Surgery, University of Naples Federico II, Naples, Italy.

# Equally Contributing Authors
§These authors jointly supervised this work
* Corresponding authors

# Supplementary Figures

Supplementary Figure 1

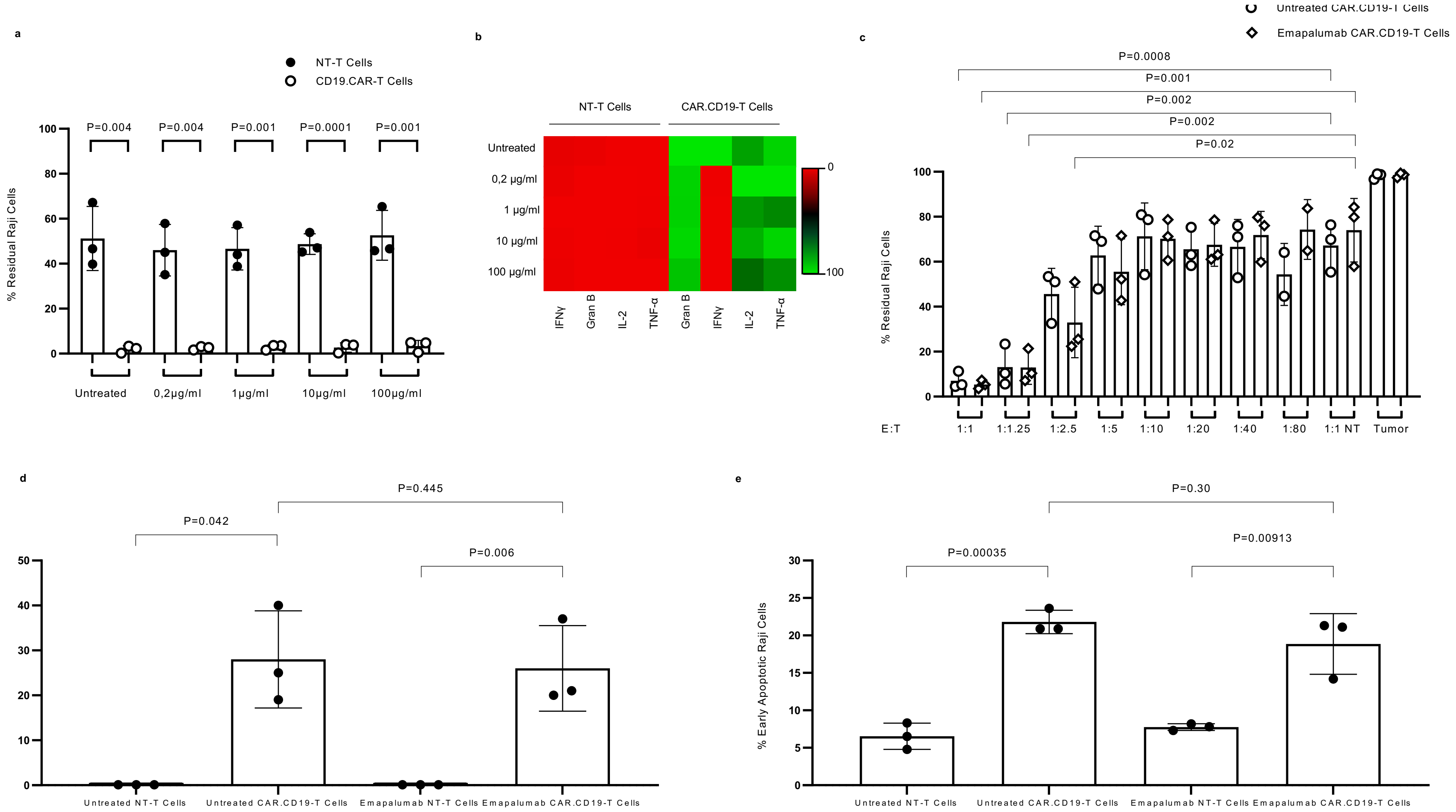

**Supplementary Figure 1. Emapalumab does not affect CAR.CD19-T cell cytotoxicity against Raji tumor cells in *in vitro* assays. (a)** NT or CAR.CD19 and GFP+ Raji cells were plated at at the 1:1 Effector (E): Target (T) ratio in media containing emapalumab within the concentration range of 0.2-100µg/ml. After 7 days, FACS analysis was performed to detect GFP+ residual tumor. Data were compared by a two-tailed Student t-test and p-value < 0.05 was considered statistically significant. **(b)** Heatmaps displaying the IFNγ, Granzyme B, IL-2 and TNF-α levels that are differentially secreted after 24h of co-culture between NT or CAR.CD19 and GFP+ Raji cells in media containing emapalumab within the concentration range of 0-100µg/ml. Colour scale represents cytokine levels as mean of 3 HDs expressed in percentage. **(c)** NT or CAR.CD19 and GFP+ Raji cells were plated at decreasing E:T ratios, from 1:1 to 1:80 in presence or absence of 100µg/ml Emapalumab. After 7 days, FACS analysis was performed to detect GFP+ residual tumor. Data were compared by a two-tailed Student t-test and p-value < 0.05 was considered statistically significant. **(d-e)** NT or CAR.CD19 and FF-LUC Raji tumor cells were plated at 1:1 Effector (E): Target (T) ratio either in the presence or in the absence of 100µg/ml Emapalumab for 6 hrs. At the end of the experiments, cells were analysed by ENSPIRE Multimode plate reader, evaluating luciferase activity **(d)**, or by FACS, evaluating Annexin V+/7AAD- tumor cells. Data were compared by a two-tailed Student t-test and p-value < 0.05 was considered statistically significant. **(e).** The data have been obtained by three independent experiments, using three different HDs for the generation of CAR.CD19-T cells. The Student's t-test was employed to evaluate the statistical relevance of differences between groups. Significative p-value (P) <0.05. Data are expressed as mean ± SD. Source data are provided as a Source Data file.

Supplementary Figure 2

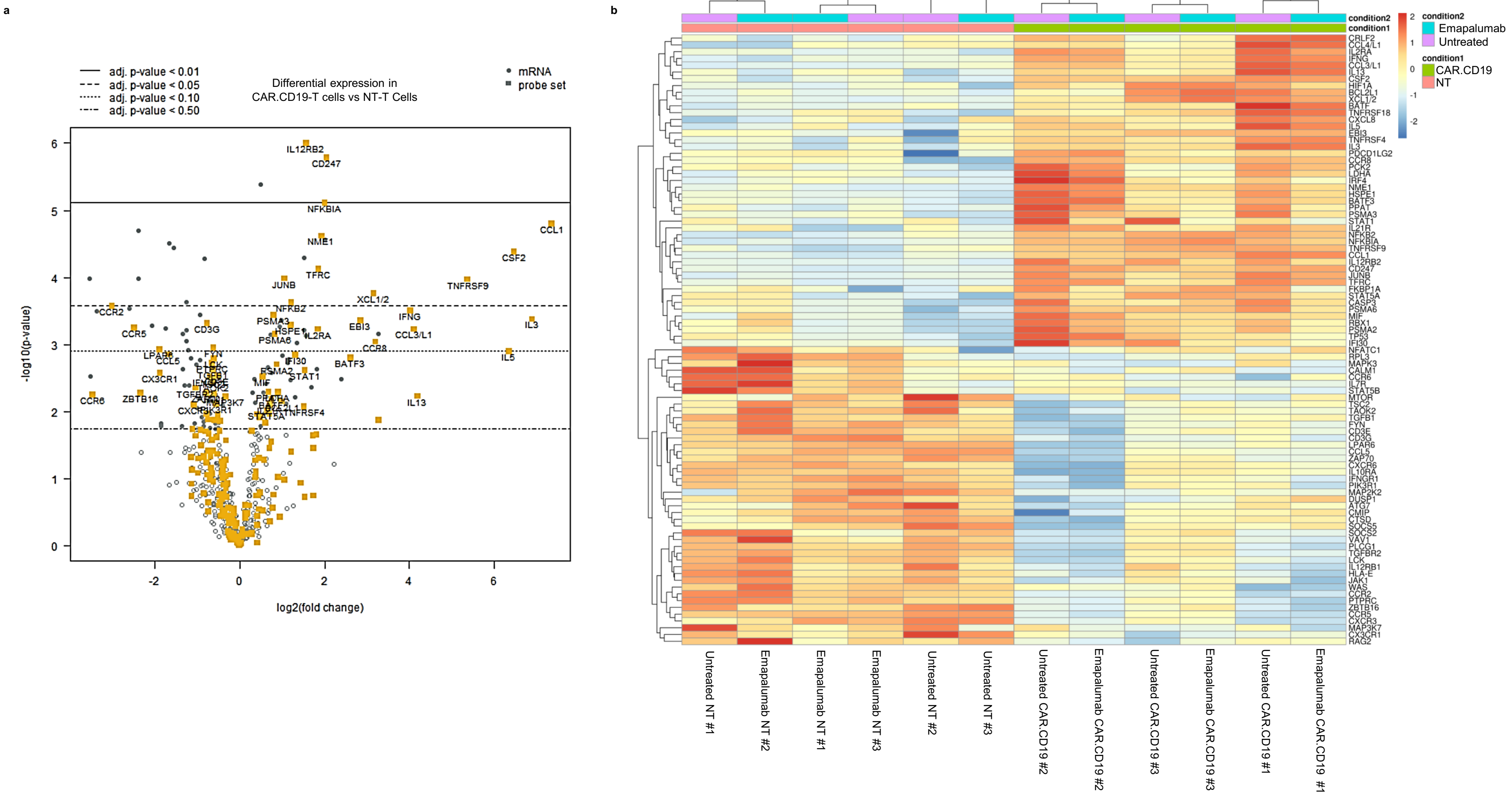

**Supplementary Figure 2. Differential expression analysis between CAR.CD19 and non-transduced (NT) T cells in either absence or presence of emapalumab.** (a) Volcano plot of modulated genes between CAR.CD19 and NT-T cells, both activated with 0,5 µg/ml Recombinant Human CD19 Fc Chimera Protein for 16 hours, not exposed to emapalumab (untreated). The log2 FC indicates the mean expression level for each gene. Genes with positive log2 FC are upregulated in activated CAR.CD19-T cells. (b) Heatmap displaying the 90 deregulated genes, all involved in activation pathways, that are differentially expressed between CAR.CD19 and NT-T cells after activation with 0,5 µg/ml Recombinant Human CD19 Fc Chimera Protein for 16 hours, in absence (untreated) or presence of 100µg/ml emapalumab. The heatmap was generated with the Pheatmap R library and the log2 transformed expression values were graphed as z-scores. Hierarchical clustering of genes and samples was applied to highlight clusters of co-regulated genes and samples with similar gene expression signatures. For differential expression analysis, a p-value of ≤0.05 (calculated with the nSolver™ 4.0 analysis software), was applied as cut-off. Source data are provided as a Source Data file.

Supplementary Figure 3

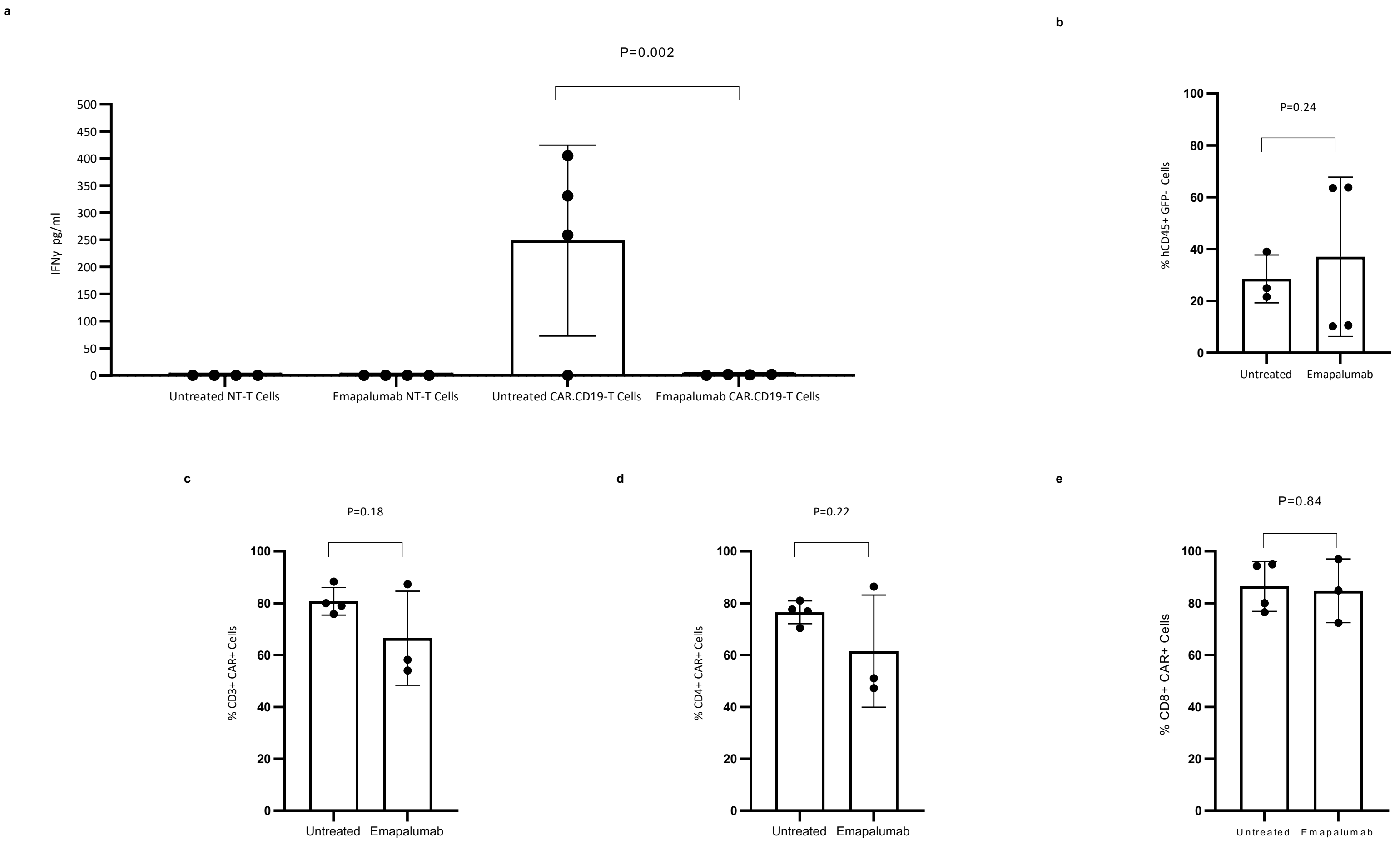

**Supplementary Figure 3. IFNγ neutralization does not affect CAR.CD19-T cell expansion in in vivo model**. **(a)** IFNγ levels were measured on peripheral blood of mice at Day+42 from effector T cell infusion. **(b-e)** Expansion of CD45+ GFP- **(b)**, CAR.CD19 CD3+ **(c)**, CAR.CD19 CD4+ **(d)**, CAR.CD19 CD8+ **(e)** cells were measured on peripheral blood of mice at Day +42 from effector T cell infusion (n=4 mice in each cohort). Data are expressed as mean ± SD. All data were compared by a two-tailed Student t-test and p-value $< 0.05$ was considered statistically significant. Source data are provided as a Source Data file.

Supplementary Figure 4

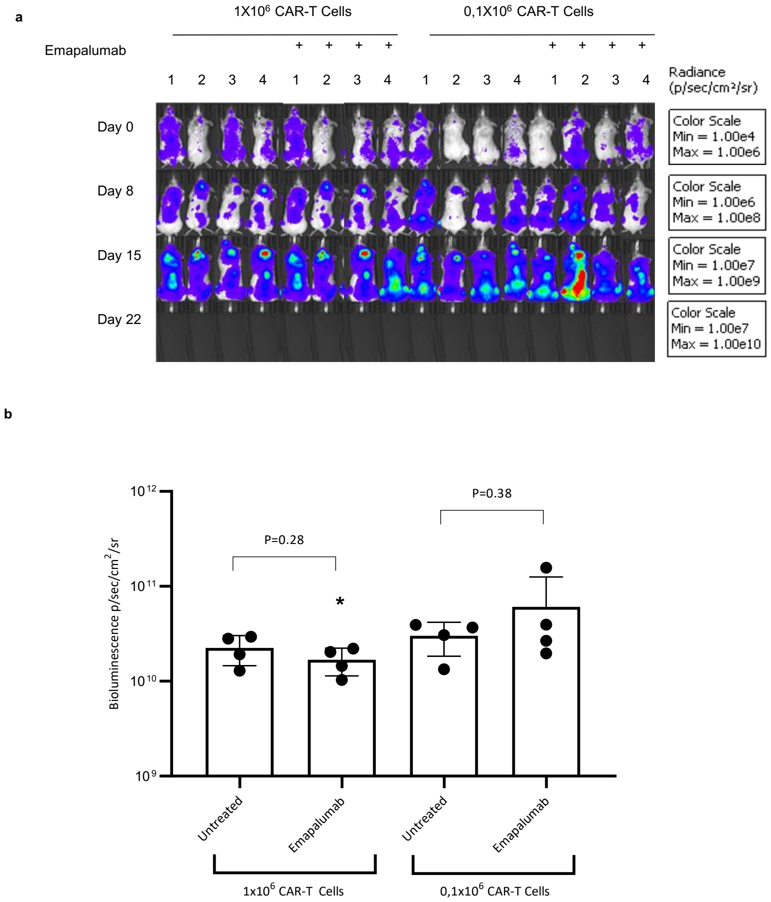

**Supplementary Figure 4. IFNγ neutralization has no impact on the cytotoxicity of CAR.CD19-T cells administered at subotimal doses. (a)** Bioluminescence imaging of mice infused with $0.25x10^6$ FF-LUC Daudi cells/mouse at Day-2 and treated with $1X10^6$ CAR.CD19-T or $0,1X10^6$ CAR.CD19-T cells/mouse at Day 0. The pre-selected cohort of mice received 100mg/Kg emapalumab at Day 7, 11, 15, and evaluated for lymphoma eradication. **(b)** Mean ± SD of bioluminescence values of mice at Day 15. The data provided in this supplementary Figure are refering to the experiment showed in Figure 5F and 5G, for which the control group of mice receiving untrasduced T cells have been included. N=4 for each cohort of treatment. Data are expressed as mean ± SD. All data were compared by a two-tailed Student t-test and p-value $< 0.05$ was considered statistically significant. * p-value=<0.05 refers to statistical significance of Emapalumab treated mice vs mice receiving untrasduced T cells. Source data are provided as a Source Data file.

Supplementary Figure 5

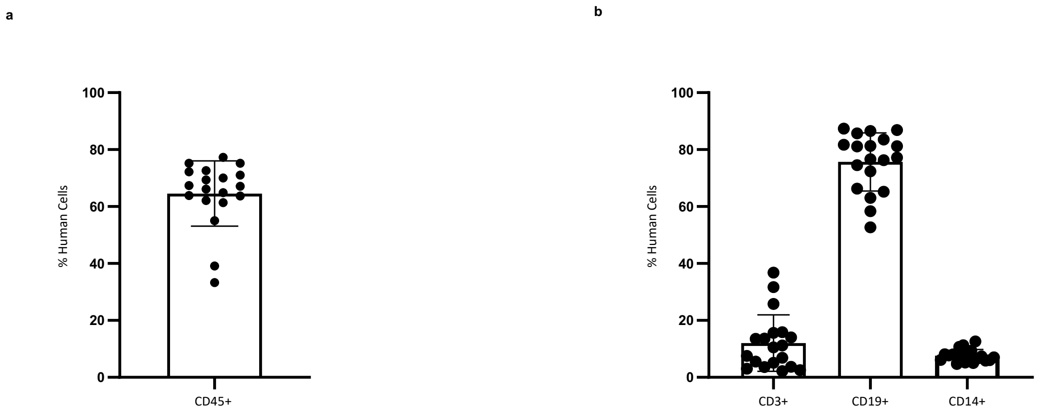


**Supplementary Figure 5. Cellular distribution of humanized mice**. Percentage of CD45+ (n=19) **(a),** CD3+, CD19+ and CD14+ (n=19) **(b)** cells were measured in peripheral blood of mice before the begin of the experiment. Data are expressed as mean ± SD. Source data are provided as a Source Data file.

Supplementary Figure 6

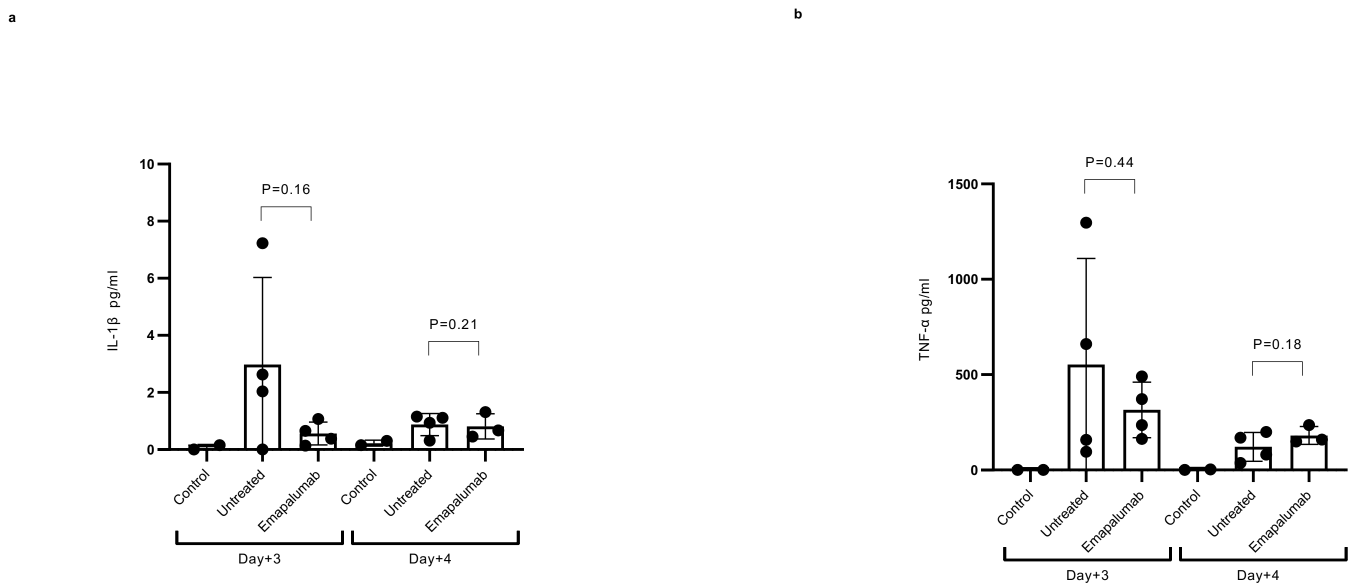


**Supplementary Figure 6. Modulation of cytokine levels by administration of emapalumab in mice developing human CRS. (a)** IL-2 and **(b)** TNF-α levels were measured in peripheral blood of negative control (n=2), treated with 10x106 CAR.CD19-T cells/mouse in presence of either 100mg/Kg/day emapalumab (n=4) or vehicle (n=4) mice at Day+3 and Day+4 from effector T cell infusion. Data are expressed as mean ± SD. All data were compared by a two-tailed Student t-test and p-value < 0.05 was considered statistically significant. Source data are provided as a Source Data file.

## Supplementary Figure 7

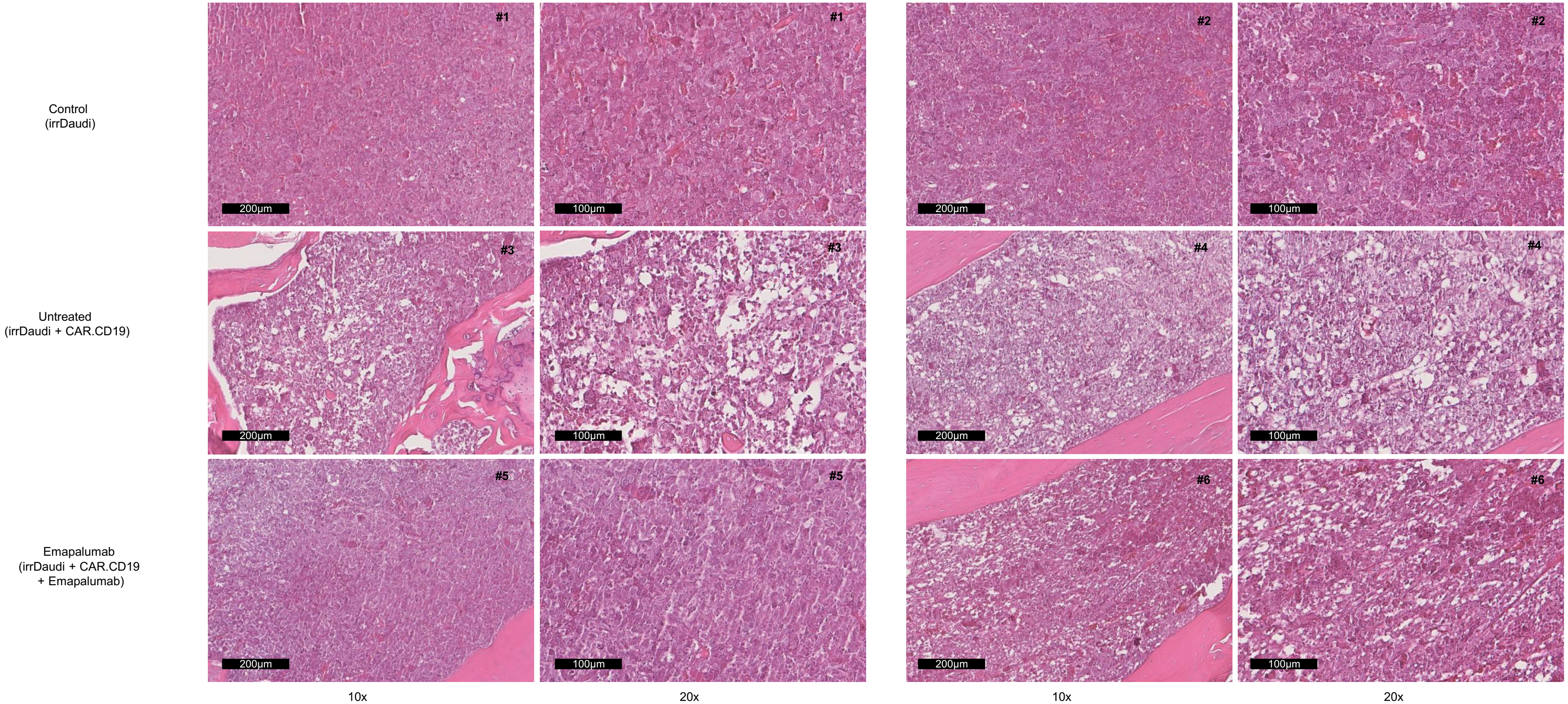

**Supplementary Figure 7. Emapalumab prevents histological bone marrow signs of macrophage activating syndrome in humanized mice developing toxicity associated to CD19.** The histopathologic haematoxylin and eosin staining was performed on bone marrow tibiae of experimental control mice engrafted only with lymphoma (#1 and #2), mice developing CRS after CAR-T cell infusion (#3 and #4), and mice treated with emapalumab (#5 and #6). IHC staining has been performed on five slides from each murine tibia.

Supplementary Figure 8

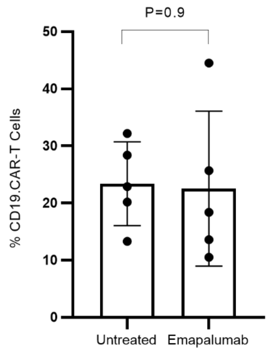


**Supplementary Figure 8. IFNγ neutralization does not affect the CAR.CD19-T cell expansion in humanized mouse model.** CAR.CD19-T cells were measured by cytofluorimetric analysis in peripheral blood of treated with $10x10^6$ CAR.CD19-T cells/mouse in presence of either 100mg/Kg/day emapalumab (n=5) or vehicle (n=5) mice at Day+3 and Day+4 after effector T cell infusion on the day of sacrifice (Day+3). Data are expressed as mean ± SD. All data were compared by a two-tailed Student t-test and p-value $< 0.05$ was considered statistically significant. Source data are provided as a Source Data file.

## Supplementary Tables

## Supplementary Table 1.

| GENE | Log2 fold change | std error (log2) | P-value | Gene.sets |
|---|---|---|---|---|
| STAT1 | -1.73 | 0.355 | 0.00124 | Activation, Innate-like T cells, JAK-STAT, Notch, Phenotype, Th1 |
| IL12RB2 | -0.553 | 0.119 | 0.00163 | Activation, Interleukin Signaling, JAK-STAT, Phenotype, Th1, Toxicity |
| TNFSF13B | -1.5 | 0.346 | 0.00251 | NF-kB, Toxicity |
| IFI35 | -0.725 | 0.178 | 0.00353 | Toxicity, Type I interferon signaling |
| STAT2 | -0.687 | 0.183 | 0.00565 | Activation, JAK-STAT, Toxicity, Type I interferon signaling |
| IRF1 | -1.05 | 0.309 | 0.00954 | Toxicity, Type I interferon signaling, Type II interferon signaling |
| OAS2 | -0.52 | 0.162 | 0.0123 | Toxicity, Type I interferon signaling, Type II interferon signaling |
| PSMA3 | -0.383 | 0.136 | 0.0224 | Activation, Antigen processing & presentation, Apoptosis, Cell Cycle, Exhaustion, Metabolism, Notch, Phenotype, Wnt signaling |
| OAS1 | -0.771 | 0.307 | 0.0363 | Toxicity, Type I interferon signaling, Type II interferon signaling |
| IFI30 | -0.685 | 0.273 | 0.0364 | Activation, Antigen processing & presentation, Toxicity, Type II interferon signaling |
| CCL28 | -0.437 | 0.175 | 0.0369 | Activation, Chemokine Signaling, Toxicity |
| ITGAM | 0.512 | 0.206 | 0.0375 | Persistence, T cell migration |
| PGK1 | -0.112 | 0.0449 | 0.0377 | Glycolysis, Metabolism |
| ADAR | -0.355 | 0.153 | 0.0487 | Toxicity, Type I interferon signaling |

**Legend Supplementary Table 1.** List of the differentially expressed genes in CAR.CD19-T cells *vs* NT-T cells, activated with 0,5 µg/ml Recombinant Human CD19 Fc Chimera Protein for 16 hours, in the presence of 100µg/ml Emapalumab. For differential expression analysis, a p-value of ≤0.05 (calculated with the nSolver™ 4.0 analysis software), was applied as cut-off. Source data are provided as a Source Data file.

## Supplementary Table 2

| GENE | Log2 fold change | std error (log2) | P-value |
|---|---|---|---|
| MAPK10 | -0.827 | 0.14 | 0.000354 |
| UBQLN2 | -0.524 | 0.133 | 0.00435 |
| SLC8A1 | -0.49 | 0.125 | 0.00446 |
| CRKL | -0.862 | 0.232 | 0.00591 |
| STAT1 | -1.44 | 0.395 | 0.00666 |
| PGK1 | -0.63 | 0.18 | 0.00822 |
| CUL1 | -1.43 | 0.43 | 0.0104 |
| PLEKHB1 | -0.403 | 0.126 | 0.0125 |
| SLC25A4 | -0.673 | 0.216 | 0.0142 |
| TXN | -0.832 | 0.267 | 0.0144 |
| TRIM46 | -1.29 | 0.418 | 0.0149 |
| GALC | -0.284 | 0.0942 | 0.0166 |
| UNC80 | -0.811 | 0.27 | 0.0168 |
| SYN2 | -0.637 | 0.212 | 0.0171 |
| CACNA2D3 | -0.34 | 0.114 | 0.0178 |
| ERBB3 | -0.788 | 0.28 | 0.0226 |
| MAPK9 | -0.519 | 0.185 | 0.0232 |
| B2M | -0.671 | 0.25 | 0.0278 |
| SLC9A6 | -0.5 | 0.191 | 0.0308 |
| RBX1 | -0.635 | 0.244 | 0.0314 |
| TRAC | -0.616 | 0.237 | 0.0315 |
| CCNH | 0.517 | 0.202 | 0.034 |
| SPARC | -0.577 | 0.226 | 0.0342 |
| ENO2 | -0.272 | 0.108 | 0.036 |
| TMSB4X | -0.547 | 0.22 | 0.0378 |
| PDPK1 | -0.54 | 0.22 | 0.0394 |
| CALM1 | -0.354 | 0.145 | 0.0405 |
| DLAT | -0.566 | 0.236 | 0.0434 |
| CNTNAP1 | -0.299 | 0.125 | 0.0435 |
| PSMC6 | -0.575 | 0.244 | 0.0462 |
| ARPC5L | -0.46 | 0.197 | 0.0482 |
| CX3CL1 | -0.838 | 0.36 | 0.0486 |
| HLA-E | -0.957 | 0.412 | 0.0489 |

**Legend Supplementary Table 2.** List of the differentially expressed genes in murine brain developing CRS with or without Emapalumab administration. For differential expression analysis, a p-value of $\leq 0.05$ (calculated with the nSolver™ 4.0 analysis software), was applied as cut-off. Source data are provided as a Source Data file.

**Supplementary Table 3**

| Antibody target/Fluorescence | Supplier Name | Cat. Number | Clone | Lot Number | Dilution |
|---|---|---|---|---|---|
| CD3 BV421 | BD Biosciences | 562426 | UCHT1 | 9113553 | 1:100 |
| CD3 APC | Immunological Sciences | MAB-02-APC | MEM-57 | 537860 | 1:100 |
| CD4 BUV496 | BD Biosciences | 612936 | SK3 | 2115049 | 1:100 |
| CD8 FITC | BD Biosciences | 555366 | RPA-T8 | 1025606 | 1:100 |
| CD14 PE | Miltenyi | 130-110-519 | REA599 | 5220402047 | 1:100 |
| CD19 BV421 | BD Biosciences | 562440 | HIB19 | 1039369 | 1:100 |
| CD25 APC | BD Biosciences | 340907 | 2A3 | 1159245 | 1:100 |
| CD28 BUV 563 | BD Biosciences | 741392 | CD28.2 | 2082945 | 1:100 |
| CD34 PE | Bio-techne s.r.l. | FAB7227P | QBEnd10 | ACOG0519121 | 1:10 |
| CD38 BUV 661 | BD Biosciences | 612969 | HIT2 | 1134609 | 1:100 |
| CD40 L APC VIO 770 | Miltenyi | 130-127-532 | REA238 | 5220609678 | 1:100 |
| CD44 BUV805 | BD Biosciences | 742019 | G44-26 | 1134609 | 1:100 |
| CD45 BUV805 | BD Biosciences | 612891 | HI30 | 1077722 | 1:100 |
| CD69 BV786 | BD Biosciences | 563834 | FN50 | 1097696 | 1:100 |
| HLA DR BUV395 | BD Biosciences | 565972 | G46-6 | 2207905 | 1:100 |
| Annexin V BUV395 | BD Biosciences | 564871 | | 1181872 | 1:100 |
| 7-Amino-Actinomycin D | BD Biosciences | 51-68981E | | 1095376 | 1:50 |

**Legend Supplementary Table 3.** List of the Antibodies used for phenotype analyses and their specific fluorescences, supplier, catalog number, clone, stock and dilution.

**Supplementary Methods**

**Gating strategy**

**Gating strategy for Figure 1a, Figure 1c, Figure 2a, Supplementary Figure 1a, Supplementary Figure 1c**

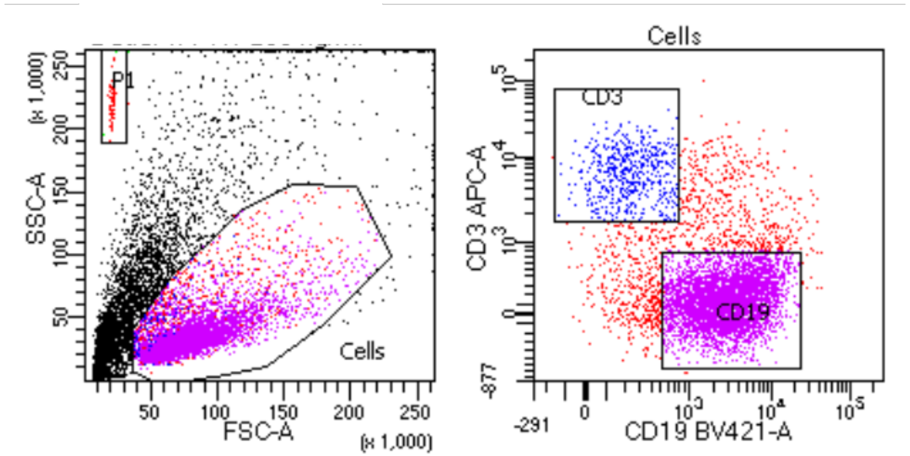

**Gating strategy for Supplementary Figure 1e**

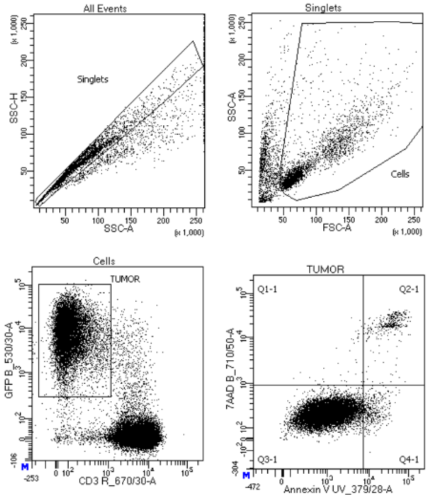

## Gating strategy for Figure 4

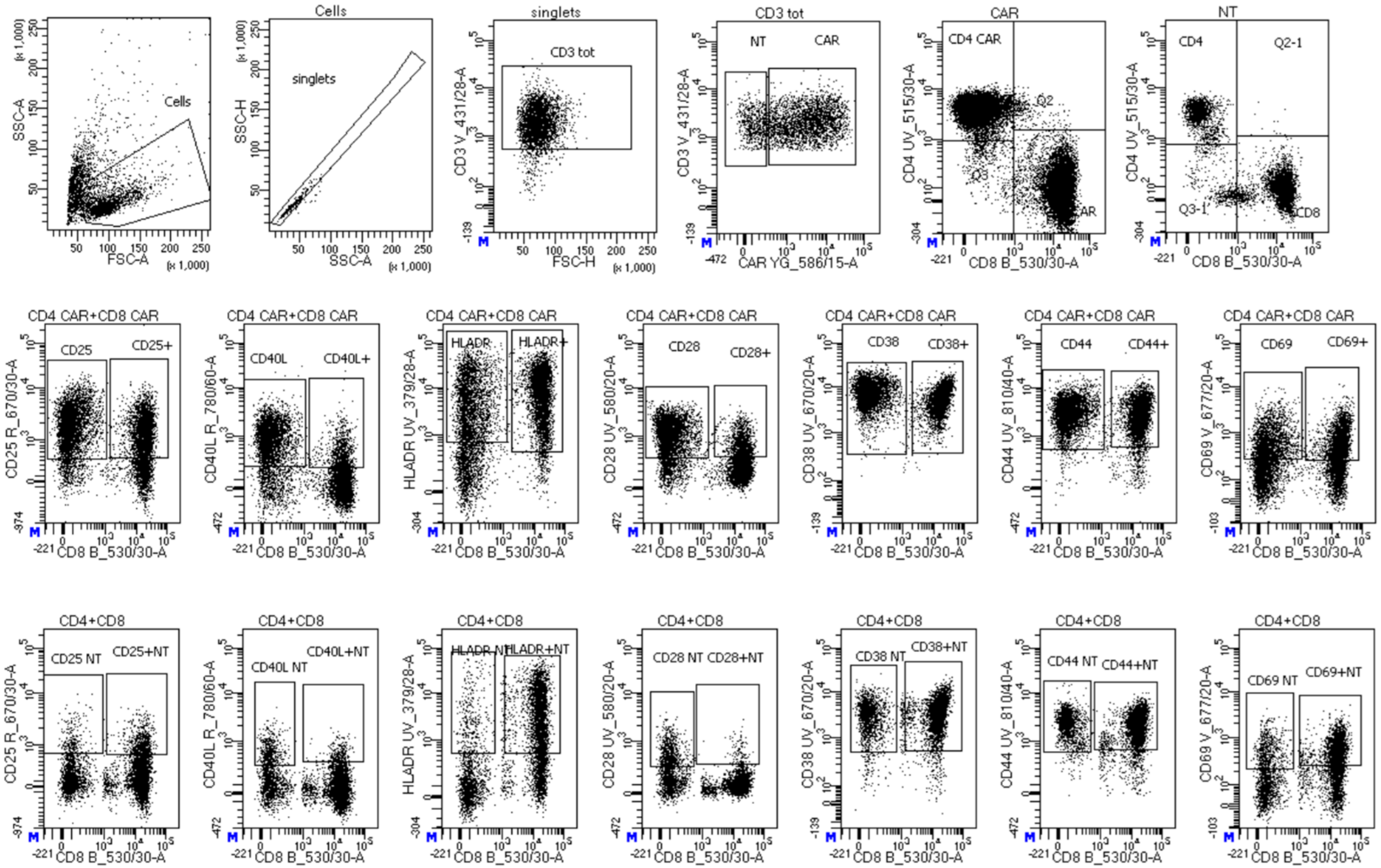

## Gating strategy for Supplementary Figure 3b-e, Supplementary Figure 8

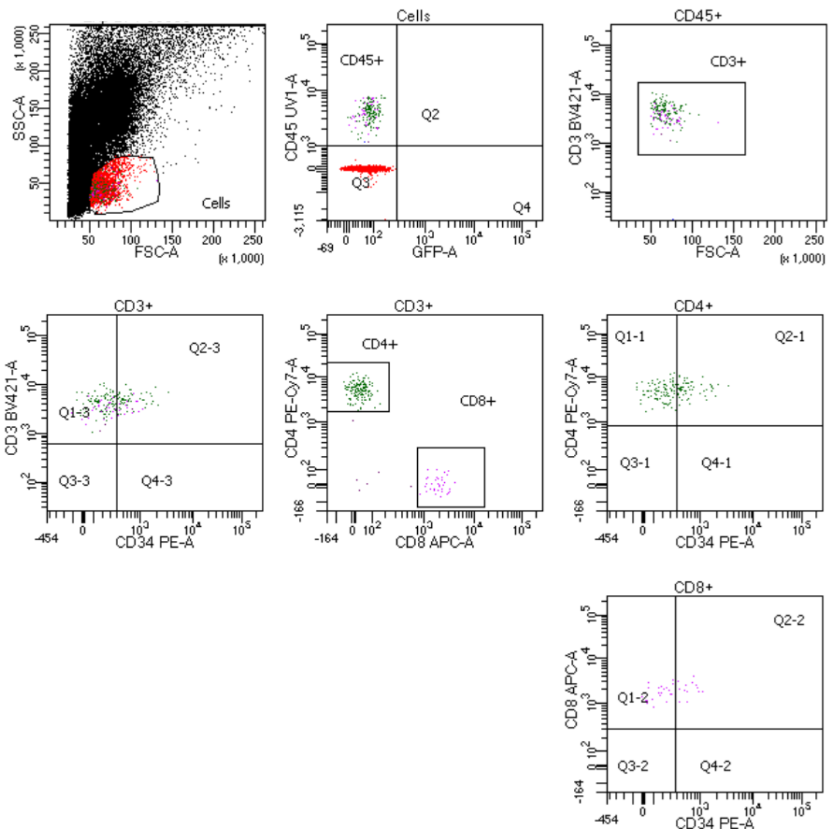

**Gating strategy for Supplementary Figure 5**

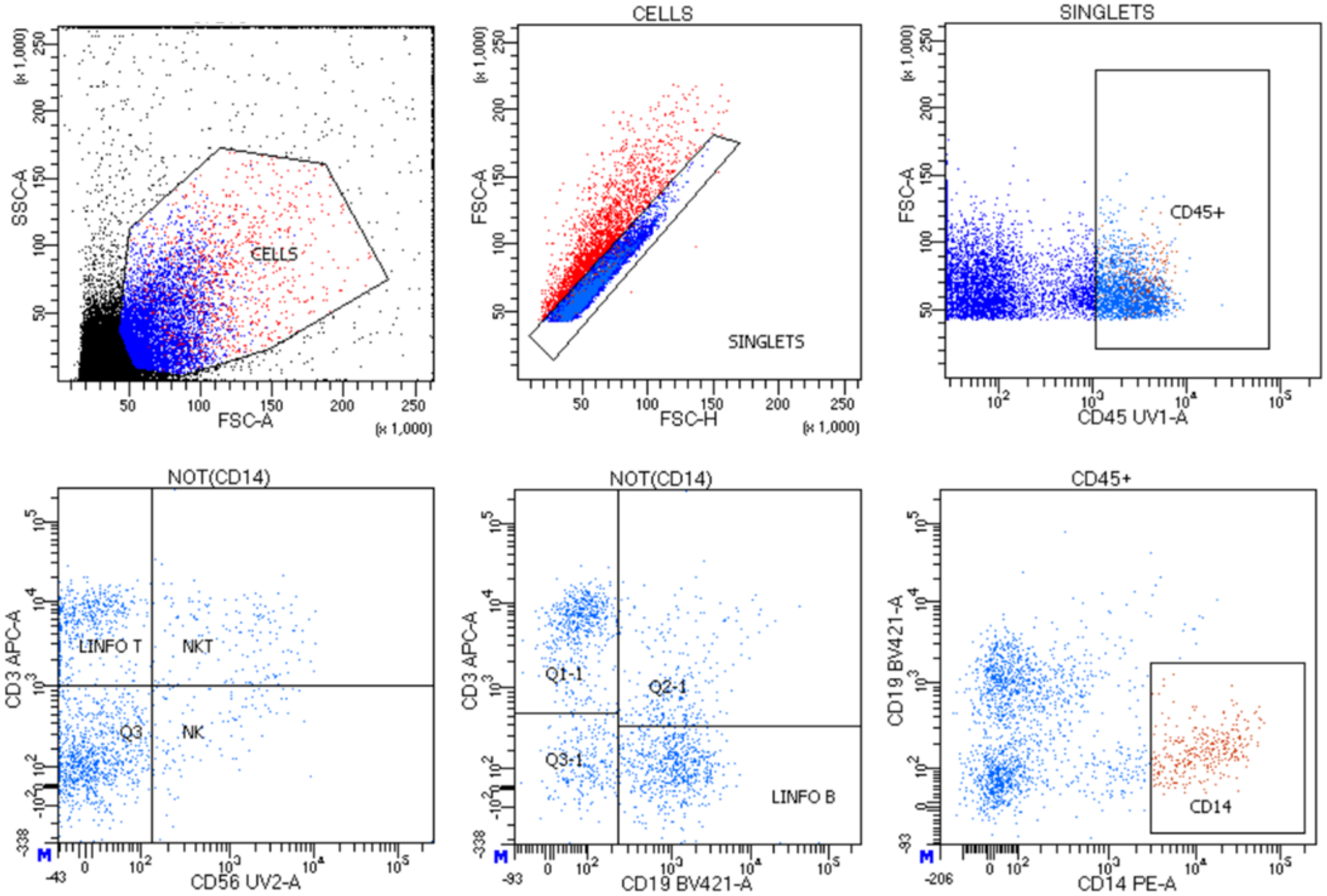

**Mouse euthanasia method**

Mice were euthanized a by inhaled gas anesthesia overdose.

**Supplementary Notes**

Declaration of the animal stocks included in the experiments:

Cg-Prkdcscid Il2rgtm1Wjl/SzJ (NSG): LOT#:614NSG

CIEA NOG-EXL: LOT#: HSCCB-13395-20210826-007; HSCCB-13395-20210331-031; HSCCB-13395-20210401-032.
