# Supplementary material for: Neutralizing IFNγ improves safety without compromising efficacy of CAR-T cell therapy in B-cell malignancies
Source: Nat Commun. 2023 Jun 9;14:3423. doi: 10.1038/s41467-023-38723-y (PMC10256701; doi:10.1038/s41467-023-38723-y)
Supplement: Supplementary file 3 — Description of Additional Supplementary Files [file 41467_2023_38723_MOESM3_ESM.pdf]

Description of Additional Supplementary Files

File Name: Supplementary Data 1
Description: **List of the differentially expressed genes belonging to activation pathway in CAR.CD19 vs untransduced T-cells (NT), in absence of Emapalumab.** For differential expression analysis, a p-value of ≤0.05 (calculated with the nSolver™ 4.0 analysis software), was applied as cut-off.

File Name: Supplementary Data 2
Description: **String annotation of modulated genes in CAR.CD19 T-cells activated with 0,5 µg/ml Recombinant Human CD19 Fc Chimera Protein for 16 hours, in the presence of 100µg/ml Emapalumab.**
