# Supplementary material for: Neutralizing IFNγ improves safety without compromising efficacy of CAR-T cell therapy in B-cell malignancies
Source: Nat Commun. 2023 Jun 9;14:3423. doi: 10.1038/s41467-023-38723-y (PMC10256701; doi:10.1038/s41467-023-38723-y)
Supplement: Supplementary file 6 — Reporting Summary [file 41467_2023_38723_MOESM6_ESM.pdf]

nature portfolio

Corresponding author(s): Franco Locatelli and Biagio De Angelis

Last updated by author(s): Apr 21, 2023

Reporting Summary

Nature Portfolio wishes to improve the reproducibility of the work that we publish. This form provides structure for consistency and transparency in reporting. For further information on Nature Portfolio policies, see our Editorial Policies and the Editorial Policy Checklist.

Statistics

For all statistical analyses, confirm that the following items are present in the figure legend, table legend, main text, or Methods section.

n/a	Confirmed	
☐	☒	The exact sample size (*n*) for each experimental group/condition, given as a discrete number and unit of measurement
☐	☒	A statement on whether measurements were taken from distinct samples or whether the same sample was measured repeatedly
☐	☒	The statistical test(s) used AND whether they are one- or two-sided<br>*Only common tests should be described solely by name; describe more complex techniques in the Methods section.*
☒	☐	A description of all covariates tested
☒	☐	A description of any assumptions or corrections, such as tests of normality and adjustment for multiple comparisons
☐	☒	A full description of the statistical parameters including central tendency (e.g. means) or other basic estimates (e.g. regression coefficient) AND variation (e.g. standard deviation) or associated estimates of uncertainty (e.g. confidence intervals)
☐	☒	For null hypothesis testing, the test statistic (e.g. *F*, *t*, *r*) with confidence intervals, effect sizes, degrees of freedom and *P* value noted<br>*Give P values as exact values whenever suitable.*
☒	☐	For Bayesian analysis, information on the choice of priors and Markov chain Monte Carlo settings
☒	☐	For hierarchical and complex designs, identification of the appropriate level for tests and full reporting of outcomes
☒	☐	Estimates of effect sizes (e.g. Cohen's *d*, Pearson's *r*), indicating how they were calculated

Our web collection on statistics for biologists contains articles on many of the points above.

Software and code

Policy information about availability of computer code

Data collection	Gene Expression data have been collected by nSolver™ 4.0 analysis software; ELISA data have been collected in ELLA Instrument by Simple Plex software (v3.9.0.28); Flow-cytometry data have been collected by FACSDiva software(v9.0.1) ; in vivo bioluminescence has been collected toward IVIS Imaging system software (4.7.3) ; IHC data have been collected toward ScanScope XT scanner and digitized to scalable images by NDPVIEW V.2 software; cytotoxicity kinetics data collected toward Incucyte [algorithm in the "2021C" software (Schrödinger; New York, NY, USA)]; Bioluminescence in vitro data collected toward Enspire Multimode plate reader (Inspire Manager software, Version 4.13.3005.1482).
Data analysis	Data analysis has been performed by GraphPad Prism 9.3.1 software; Gene Expression data analysis has been performed toward nCounter® Digital Analyzer and nSolver™ 4.0 analysis software

For manuscripts utilizing custom algorithms or software that are central to the research but not yet described in published literature, software must be made available to editors and reviewers. We strongly encourage code deposition in a community repository (e.g. GitHub). See the Nature Portfolio guidelines for submitting code & software for further information.

Data

Policy information about availability of data

All manuscripts must include a data availability statement. This statement should provide the following information, where applicable:

- Accession codes, unique identifiers, or web links for publicly available datasets
- A description of any restrictions on data availability
- For clinical datasets or third party data, please ensure that the statement adheres to our policy

All data generated or analysed during this study are included in this manuscript (and its supplementary information files).

Human research participants

Policy information about studies involving human research participants and Sex and Gender in Research.

Reporting on sex and gender	Sex and gender information was not collected because sex and gender do not affect production and effectiveness of CAR T-cells.
Population characteristics	Characteristics of the human research participants (e.g. age) were not collected in this study. Healthy donors were not screened for any covariate-relevant population characteristics, since they have been enrolled only to provide peripheral blood for the production of CAR-T cells, for which no relevant population characteristics are expected to have impact.
Recruitment	Participants are healthy donors recruited from the Children's Hospital Bambino Gesu who have signed informed consent, without discrimination for any kind including gender, agee, race, nationality, prospective donors' health status and medical history.
Ethics oversight	The handling of human samples was conducted in accordance with the Institutional Review Board (IRB) of Bambino Gesù Children's Hospital, IRCCS, Rome, Italy (OPBG; Ethics Committee Approval N°969/2015 prot.N°669LB, and N°1422/2017 prot.N°810).

Note that full information on the approval of the study protocol must also be provided in the manuscript.

Field-specific reporting

Please select the one below that is the best fit for your research. If you are not sure, read the appropriate sections before making your selection.

☒ Life sciences ☐ Behavioural & social sciences ☐ Ecological, evolutionary & environmental sciences

For a reference copy of the document with all sections, see nature.com/documents/nr-reporting-summary-flat.pdf

Life sciences study design

All studies must disclose on these points even when the disclosure is negative.

Sample size	We estimated the sample size considering no significant variation within each group of data. The principle of using the smallest sample size possible was adopted in planning the animal experiments. We estimated the sample size in order to detect a difference in averages of 2 standard deviations at the 0.05 level of significance with an 80% power.
Data exclusions	No data were excluded from any analysis included in our results.
Replication	The experiments were repeated as described in the figure captions, achieving overlapping results, all reported in the figures of the manuscript.
Randomization	Experimental mice were randomized into the experimental groups following tumor inoculation before CAR-T cell infusion according to bioluminescence tumor value to ensure equal mean tumor burden per condition at the start of treatment. For studies not involving animals, no randomization was required.
Blinding	In in vivo studies, blinding were performed only at the start of the experiments, when mice were randomized into the experimental groups following tumor inoculation before CAR-T cell infusion. Moreover, the bio-lumiscence data were collected in a blinding manner. For all the other subsequent steps, blinding was not feasible given the study design and the labeling requirements for the operative procedures of the facilities. IHC stainings were performed in blinding manner.

Reporting for specific materials, systems and methods

We require information from authors about some types of materials, experimental systems and methods used in many studies. Here, indicate whether each material, system or method listed is relevant to your study. If you are not sure if a list item applies to your research, read the appropriate section before selecting a response.

Materials & experimental systems

n/a	Involved in the study
☐	☒ Antibodies
☐	☒ Eukaryotic cell lines
☒	☐ Palaeontology and archaeology
☐	☒ Animals and other organisms
☒	☐ Clinical data
☒	☐ Dual use research of concern

Methods

n/a	Involved in the study
☒	☐ ChIP-seq
☐	☒ Flow cytometry
☒	☐ MRI-based neuroimaging

Antibodies

Antibodies used

1) CD3 BV421: Supplier Name: BD Biosciences; Clone: UCHT1; Cat Number:562426; Clone: UCHT1; Lot Number: 9113553; Dilution 1:100.
2) CD3 APC: Supplier Name: Immunological Sciences; Cat Number: MAB-02-APC; Clone: MEM-57; Lot Number: 537860; Dilution 1:100.
3) CD4 BUV496: Supplier Name: BD Biosciences; Cat Number: 612936; Clone: SK3; Lot Number:2115049; Dilution 1:100.
4) CD8 FITC: Supplier Name: BD Biosciences; Cat Number: 555366; Clone: RPA-T8; Lot Number: 1025606; Dilution 1:100.
5) CD14 PE: Miltenyi; Cat Number: 130-110-519; Clone: REA599; Lot Number:5220402047; Dilution 1:100.
6) CD19 BV421: Supplier Name: BD Biosciences; Cat Number: 562440; Clone: HIB19; Lot Number: 1039369; Dilution 1:100.
7) CD25 APC: Supplier Name: BD Biosciences; Cat Number: 340907; Clone: 2A3 Lot Number:1159245; Dilution 1:100.
8) CD28 BUV 563: Supplier Name: BD Biosciences; Cat Number: 741392; Clone: CD28.2; Lot Number: 2082945; Dilution 1:100.
9) CD34 Pe: Supplier Name: Bio-techne s.r.l.: Cat Number: FAB7227P; Clone: QBEnd10; Lot Number: ACOG0519121; Dilution 1:10.
10) CD38 BUV 661: Supplier Name: BD Biosciences; Cat Number: 612969; Clone: HIT2; Lot Number: 1134609; Dilution 1:100.
11) CD40 L APC VIO 770: Supplier Name: Miltenyi; Cat Number: 130-127-532; Clone: REA238; Lot Number: 5220609678 ; Dilution 1:100.
12) CD44 BUV805: Supplier Name: BD Biosciences; Cat Number: 742019; Clone: G44-26; Lot Number:1134609; Dilution 1:100.
13) CD45 BUV805: Supplier Name: BD Biosciences; Cat Number: 612891; Clone:HI30; Lot Number:1077722; Dilution 1:100.
14) CD69 BV786: Supplier Name: BD Biosciences; Cat Number: 563834; Clone: FN50; Lot Number:1097696 ; Dilution 1:100.
15) HLA DR BUV395: Supplier Name: BD Biosciences; Cat Number: 565972; Clone: G46-6; Lot Number:2207905; Dilution 1:100.
16) Annexin V BUV395: Supplier Name: BD Biosciences; Cat Number: 564871; Lot Number: 1181872; Dilution 1:100.
17) 7-Amino-Actinomycin D: Supplier Name: BD Biosciences; Cat Number: 51-68981E; Lot Number: 1095376; Dilution 1:50.

Validation

Each antibody used in this study has been validated by its manufacture, i.e. BD biosciences, immunological sciences, Miltenyi, Bio-techne.

Eukaryotic cell lines

Policy information about cell lines and Sex and Gender in Research

Cell line source(s)

CD19 positive human Burkitt's lymphoma cell lines Daudi and Raji were purchased by from American Type Culture Collection Company (ATCC, USA).

Authentication

All cell lines were authenticated by PCR-single-locus-technology (Promega, USA. PowerPlex 21 PCR) analysis in "BMR Genomics s.r.l." (Italy).

Mycoplasma contamination

All cells were confirmed to be negative for mycoplasma by PCR as described in the manuscript after every freeze-thaw cycle and before injection into mice.

Commonly misidentified lines (See ICLAC register)

In our paper, we have not applied the use of misidentified lines

Animals and other research organisms

Policy information about studies involving animals; ARRIVE guidelines recommended for reporting animal research, and Sex and Gender in Research

Laboratory animals

1) Species: Mus musculus; strain: Cg-Prkdcscid Il2rgtm1Wjl/SzJ (NSG provided by Charles River); sex: female; Age: 6 weeks (for anti-leukemia murine model).

	2) Species: Mus musculus; strain: CIEA NOG-EXL (provided by Taconic); sex: female; age: 14 weeks (for humanized murine model).<br><br>Mice were bred under specific pathogen-free conditions. Mice were maintained in a controlled sterile environment (gradual light-dark cycle with light from 7 a.m. to 5 p.m., 21-25°C, 45-65% humidity).
Wild animals	In our paper, we have not applied the use of wild animals
Reporting on sex	1) Species: Mus musculus; strain: Cg-Prkdcscid Il2rgtm1Wjl/SzJ (NSG provided by Charles River); sex: female;<br>2) Species: Mus musculus; strain: CIEA NOG-EXL (provided by Taconic); sex: female;
Field-collected samples	In our paper, we have not applied the use of field-collected samples
Ethics oversight	All procedures were performed in accordance with the Guidelines for Animal Care and Use of the National Institutes of Health (Ethical committee for animal experimentation Prot. N 088/2016-PR).

Note that full information on the approval of the study protocol must also be provided in the manuscript.

Flow Cytometry

Plots

Confirm that:

☒ The axis labels state the marker and fluorochrome used (e.g. CD4-FITC).

☒ The axis scales are clearly visible. Include numbers along axes only for bottom left plot of group (a 'group' is an analysis of identical markers).

☒ All plots are contour plots with outliers or pseudocolor plots.

☒ A numerical value for number of cells or percentage (with statistics) is provided.

Methodology

Sample preparation	Flow-cytometry analysis was performed to determine cell surface antigen expression; monoclonal antibodies were combined with different fluorescence according to needs. CAR.CD19 expression was detected using a mAb directed to hCD34 epitope (anti-CD34 QBend-10 PE from Bio-Techne, MN, USA). Cells were collected from co-coltures, washed with PBS and incubated with the antibodies as indicated by the manufacturer for 30 minutes at +4°C. Afterwards, cells were washed in PBS and acquired to the flow cytometer.
Instrument	Flow-cytometry analysis was performed using a BD LSRFortessa X-20 cytometer (BD Biosciences, USA) and FACSymphony™ A5(BD Biosciences, USA).
Software	Flow-cytometry analysis was analyzed by FACSDiva software (BD Biosciences, USA)
Cell population abundance	GFP positive cells have been FACS-sorted to achieve 100% of positive cells expressing GFP as well as FF_Luciferase. After sorting, the cells have been expanded and checked for GFP expression before any further application, both in vitro and in vivo.
Gating strategy	The gating strategy for Figure 1a, Figure 1c, Figure 2a, Supplemental Figure 1a, Supplemental Figure 1c:<br>1) in the forward scatter and side scatter (FSC-A/SSC-A) dot plot selection of the lymphocyte gate;<br>2) in the lymphocyte gate, opening of GFP (as tumor) and CD3 (as T cells) parameters.<br><br>The gating strategy for Figure 4:<br>1) in the forward scatter and side scatter (FSC-A/SSC-A) dot plot selection of the lymphocyte gate;<br>2) in the lymphocyte gate, opening of SSC-H and SSC-A to select singlets;<br>3) in the singlets gate, opening of CD3 (as T cells) and CD34 (as for CAR+ T cells) parameters;<br>4) in the CD3+ gate, opening CD8 and CD4 parameters for the NT T-cell population;<br>5) in the CD34+ gate, opening CD8 and CD4 parameters for the CAR+ T-cell population;<br>6) in the CD3+ CD4+ and CD3+ CD8+gates, opening of CD25, CD40L, HLA-DR, CD28, CD38, CD44 and CD69 vs CD8 parameters (for NT T cell condition);<br>6) in the CD34+ CD4+ and CD34+ CD8+gates, opening of CD25, CD40L, HLA-DR, CD28, CD38, CD44 and CD69 vs CD8 parameters (for CAR.CD19 T cell condition);<br><br>The gating strategy for Supplemental Figure 1e:<br>1) opening of SSC-H and SSC-A to select singlets;<br>2) in the singlets gate opening the forward scatter and side scatter (FSC-A/SSC-A) dot plot to select the lymphocyte gate;<br>3) in the lymphocyte gate, opening of GFP (as tumor) and CD3 (as T cells) parameters;<br>4) in the GFP+ gate, opening of 7AAD and Annexin V parameters.<br><br>The gating strategy for Supplemental Figure 3b-e, Supplemental Figure 7:<br>5) in the forward scatter and side scatter (FSC-A/SSC-A) dot plot selection of the lymphocyte gate;<br>6) in the lymphocyte gate, opening of human CD45 and GFP (for tumor detection) parameters;<br>7) in the CD45+ GFP- gate, opening of CD3 and FSC-A parameters;<br>8) in the CD3+ gate, opening of CD4 and CD8 parameters;

9) in the CD4+ gate, opening CD4 and CD34 (as CD4+ CAR+ T cells);
10) in the CD8+ gate, opening CD8 and CD34 (as CD8+ CAR+ T cells);

The gating strategy for Supplemental Figure 5:
1) in the forward scatter and side scatter (FSC-A/SSC-A) dot plot selection of the lymphocyte gate;
2) in the lymphocyte gate, opening of SSC-H and SSC-A to select singlets;
3) in the singlets gate, opening of CD45 and FSC-A parameters;
4) in the CD45+ gate, opening of CD14 and CD45 parameters;
5) in the CD45+ CD14- gate, opening of CD3 and CD56 parameters;
6) in the CD45+ CD14- gate, opening of CD3 and CD19 parameters.

☒ Tick this box to confirm that a figure exemplifying the gating strategy is provided in the Supplementary Information.
